# Nucleoside reverse transcriptase inhibitors and Kamuvudines inhibit amyloid-β induced retinal pigmented epithelium degeneration

**DOI:** 10.1038/s41392-021-00537-z

**Published:** 2021-04-14

**Authors:** Siddharth Narendran, Felipe Pereira, Praveen Yerramothu, Ivana Apicella, Shao-bin Wang, Kameshwari Ambati, Shuichiro Hirahara, Younghee Kim, Meenakshi Ambati, Vidya L. Ambati, Peirong Huang, Akhil Varshney, Yosuke Nagasaka, Shinichi Fukuda, Kirstie L. Baker, Kenneth M. Marion, Jan M. Deussing, Srinivas R. Sadda, Bradley D. Gelfand, Jayakrishna Ambati

**Affiliations:** 1 Center for Advanced Vision Science, University of Virginia School of Medicine, Charlottesville, VA USA; 2 Department of Ophthalmology, University of Virginia School of Medicine, Charlottesville, VA USA; 3 Aravind Eye Care System, Madurai, India; 4 Departamento de Oftalmologia e Ciências Visuais, Escola Paulista de Medicina, Universidade Federal de São Paulo, São Paulo, Brazil; 5 Center for Digital Image Evaluation, Charlottesville, VA USA; 6 Doheny Eye Institute, Los Angeles, Los Angeles, CA USA; 7 Molecular Neurogenetics, Department of Stress Neurobiology and Neurogenetics, Max Planck Institute of Psychiatry, Munich, Germany; 8 Department of Ophthalmology, David Geffen School of Medicine, University of California–Los Angeles, Los Angeles, CA USA; 9 Department of Biomedical Engineering, University of Virginia School of Medicine, Charlottesville, VA USA; 10 Department of Pathology, University of Virginia School of Medicine, Charlottesville, VA USA; 11 Department of Microbiology, Immunology, and Cancer Biology, University of Virginia School of Medicine, Charlottesville, VA USA

**Keywords:** Innate immunity, Translational research

## Abstract

Nonfibrillar amyloid-β oligomers (AβOs) are a major component of drusen, the sub-retinal pigmented epithelium (RPE) extracellular deposits characteristic of age-related macular degeneration (AMD), a common cause of global blindness. We report that AβOs induce RPE degeneration, a clinical hallmark of geographic atrophy (GA), a vision-threatening late stage of AMD that is currently untreatable. We demonstrate that AβOs induce activation of the NLRP3 inflammasome in the mouse RPE in vivo and that RPE expression of the purinergic ATP receptor P2RX7, an upstream mediator of NLRP3 inflammasome activation, is required for AβO-induced RPE degeneration. Two classes of small molecule inflammasome inhibitors—nucleoside reverse transcriptase inhibitors (NRTIs) and their antiretrovirally inert modified analog Kamuvudines—both inhibit AβOs-induced RPE degeneration. These findings crystallize the importance of P2RX7 and NLRP3 in a disease-relevant model of AMD and identify inflammasome inhibitors as potential treatments for GA.

## Introduction

Geographic atrophy (GA), an irreversible and untreatable form of dry age-related macular degeneration (AMD), causes blindness in millions of individuals worldwide.^1^ Extracellular deposit external to the retinal pigmented epithelium (RPE) termed drusen are hallmark pathological features of AMD. Pro-inflammatory components present in drusen are thought to drive inflammation and disease progression in GA.^2^ Amyloid-β oligomers (AβOs) within drusen are linked to RPE degeneration in GA.^3–5^ Inhibiting AβOs-induced neuroinflammation has been a therapeutic strategy pursued amyloid-driven neurodegenerative diseases.^6^ Thus, identifying molecular sensors and inflammatory pathways mediating AβOs-induced RPE degeneration could provide insights to aid in developing GA therapies.

Inflammasomes are multimeric cytosolic protein complexes that recognize unique pathogen-associated molecular patterns (PAMPs) or damage-associated molecular patterns (DAMPs) and trigger innate immune responses by activating caspase-1-dependent cytokine production and cell death.^7^ AβOs induce activation of the NLR family pyrin domain containing 3 (NLRP3) inflammasome in neurodegenerative disease models.^8–10^ NLRP3 inflammasome activation is an indispensable driver of RPE degeneration in other models of GA.^11–13^ Therefore, inhibition of NLRP3 activation is a promising approach to halt or delay RPE degeneration and disease progression in GA.

NLRP3 inflammasome activation generally comprises a two-step process in which both the initial priming and concomitant activating signals are required to produce a functional inflammasome.^14^ Transcriptional upregulation of NLRP3, a predominant facet of priming, is an essential step, especially in non-immune cells such as the RPE because basal expression levels are considered insufficient to initiate inflammasome assembly. NLRP3 inflammasome activation also requires the assembly of a multi-protein complex that recruits pro-caspase-1 via the adapter protein ASC (an apoptosis-associated speck-like protein containing a caspase recruitment domain). ASC polymerization, which is necessary for NLRP3 inflammasome activation,^15^ is characterized by the formation of large intracellular aggregates termed ASC specks, which are a signature of inflammasome assembly. Upon assembly, pro-caspase-1 is cleaved to caspase-1, which in turn cleaves the cytokine precursors pro-IL-1β and pro-IL-18 into mature IL-1β and IL-18, which are cytotoxic to the RPE.^14^

The purinergic P2X7 receptor (P2RX7) is an essential mediator necessary for NLRP3 activation in several systems.^16^ P2RX7 has been identified as a putative drug target in several models of GA.^17–20^ AβOs-induced NLRP3 activation requires P2RX7 expression in microglial cells.^21^ AβOs-induced RPE cytotoxicity has been recently reported to be driven by mitochondrial dysfunction and reactive oxygen species (ROS),^22^ well-characterized inducers of NLRP3 inflammasome activation.^23^ Despite multiple studies reporting AβOs-induced NLRP3 activation in RPE,^24–26^ the role of P2RX7 remains unclear. Therefore, we tested if P2RX7 expression is necessary for AβOs-induced RPE degeneration.

Nucleoside reverse-transcriptase inhibitors (NRTIs) possess intrinsic anti-inflammatory activity independent of their anti-retroviral function by virtue of their ability to inhibit P2RX7 and subsequent NLRP3 activation.^17^ Repurposing NRTIs has been suggested as a possible treatment strategy for several chronic diseases.^17,27,28^ However, the toxicities associated with systemic NRTI use, which is attributed to their off-target inhibition of cellular polymerases, reduces enthusiasm for such therapeutic ventures.^29,30^ Interestingly, the anti-inflammatory function of NRTIs is independent of their ability to inhibit reverse transcriptase. Modified NRTIs known as Kamuvudines, which retain the ability to inhibit inflammasome activation but lack the ability to inhibit reverse transcriptase and hence also lack the attendant toxicities, represent better candidates for treating P2RX7-NLRP3 mediated diseases.^17,27^ Therefore, we investigated the efficacy of NRTIs and Kamuvudines to inhibit AβOs-induced RPE degeneration.

Here, we demonstrate that P2RX7 is an indispensable component necessary for AβOs-induced RPE degeneration and its inhibition by NRTIs and Kamuvudines blocks AβOs-induced RPE degeneration.

## Results

### AβOs induce NLRP3 inflammasome assembly in the RPE

**Fig. 1:**
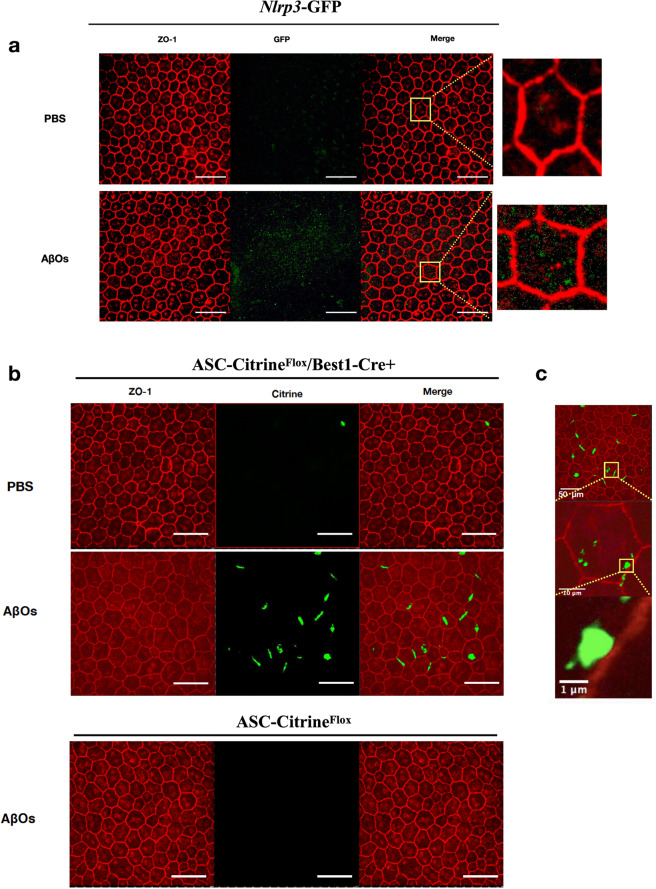
AβOs promote NLRP3 inflammasome priming and assembly in the RPE. **a** RPE flat mounts, stained for zonula occludens-1 (ZO-1; red), of *Nlrp3*-GFP knock-in mice injected subretinally with AβOs show increased GFP expression (green) compared to RPE flat mounts of saline-injected mice, *n* = 6. **b** Subretinal injection of AβOs, but not PBS, induced increased ASC speck formation in ASC-Citrine^Flox^/Best1-Cre+ mice. ASC-Citrine fusion proteins are detected as a green signal. Subretinal injection of AβOs did not induce ASC speck formation in ASC-Citrine^Flox^ mice, *n* = 6. **c** Higher magnification images of the observed ASC speck in RPE flat mounts of ASC-Citrine^Flox^/Best1-Cre+ mice injected with subretinal AβOs demonstrate the size of the specks. Selected areas of interest highlighted by the yellow squares depict the magnified regions. Scale bars (50 μm)

We monitored NLRP3 inflammasome priming in the RPE in vivo following subretinal injection of AβOs by using *Nlrp3-*GFP mice, a reporter mouse line in which transcription of the fluorescent reporter is controlled by endogenous *Nlrp3* regulatory elements.^31^ Insertion of GFP in the NLRP3 locus renders these mice functionally deficient in NLRP3. Notwithstanding this disruption in NLRP3 protein expression, we also treated these mice with an intravitreous injection of Ac-YVAD-fmk, a caspase-1 inhibitor, to eliminate any residual inflammasome due to potential leakiness. This enabled us to visualize GFP signals free of distortions arising from potential degenerating cells. Following subretinal injection of AβOs in *Nlrp3-*GFP mice, confocal microscopic analysis of RPE flat mounts revealed increased GFP expression in AβOs-injected eyes compared to control-injected eyes (Fig. 1a), providing in situ evidence of NLRP3 priming in the RPE since GFP expression in these transgenic mice expression correlates with increased NLRP3 mRNA and protein expression.^32,33^

Next, we monitored inflammasome assembly in the RPE in vivo by using ASC-Citrine^Flox^/Best1-Cre+ mice, which we generated by interbreeding ASC-Citrine^Flox^ mice, a reporter mouse model that displays fluorescent ASC specks signifying inflammasome assembly,^34^ with Best1-Cre+ mice, which express Cre specifically in the RPE. Following subretinal injection of AβOs, ASC-Citrine^Flox^/Best1-Cre+ mice displayed increased formation of 1–2 μm ASC specks in the RPE compared to control-treated mice (Fig. 1b, c). The ASC specks were restricted to RPE cells expressing Cre (Supplementary Fig. 1).

### NLRP3 inflammasome is necessary for AβOs-induced RPE degeneration

**Fig. 2:**
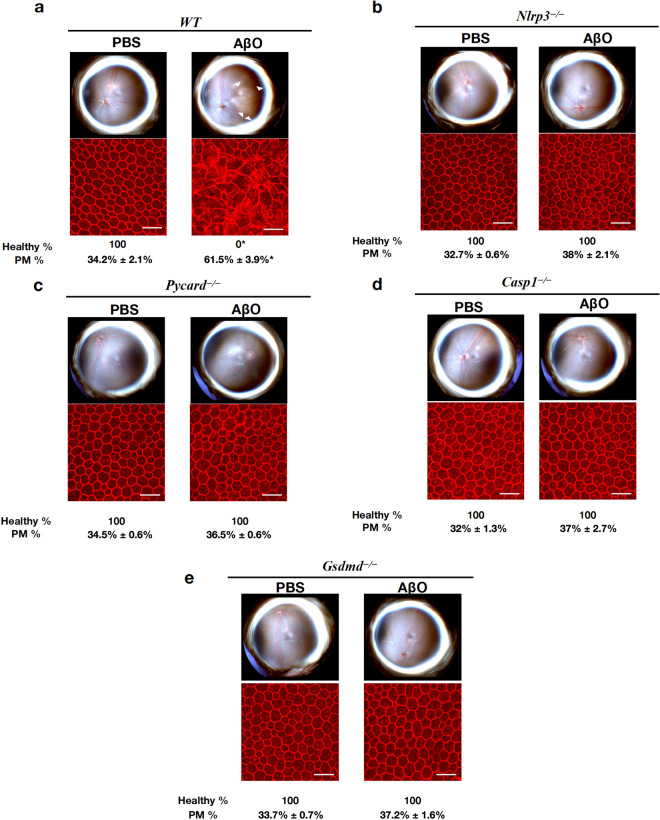
AβOs-induced RPE degeneration is NLRP3 inflammasome dependent. Eyes were treated with a single subretinal injection of 1 μM AβOs. Tissue was collected 7 days after injection. **a**–**e** AβOs induced degeneration in WT mice, *n* = 8 (**a**) but not in *Nlrp3*^*−/−*^, *n* = 8 (**b**), *Pycard*^*−/−*^, *n* = 8 (**c**), *Casp1*^*−/−*^, *n* = 8 (**d**) or *Gsdmd*^*−/−*^, *n* = 8 (**e**) mice. Fundus photographs, top row; Flat mounts stained for zonula occludens-1 (ZO-1; red), bottom row. Degeneration outlined by white arrowheads. Binary (Healthy %) and morphometric (PM, polymegethism (mean (SEM)) quantification of RPE degeneration is shown (Fisher’s exact test for binary; two-tailed *t*-test for morphometry; *P < 0.001). Loss of regular hexagonal cellular boundaries in ZO-1 stained flat mounts is indicative of degenerated RPE. Scale bars (50 μm)

Subretinal injection of AβOs of the 1–40 peptide, but not of the control reverse peptide 40-1, induced RPE degeneration in wild-type (WT) mice (Supplementary Fig 2); however, mice deficient in the inflammasome components NLRP3, gasdermin D (encoded by *Gsdmd*), caspase-1 (encoded by *Casp1*), or ASC (encoded by *Pycard*) were protected from AβOs-induced RPE degeneration (Fig. 2a–e and Supplementary Fig. 3). Morphometric analysis of the RPE flat mounts revealed significantly higher (P < 0.001) polymegethism in WT mice (61.5% ± 3.9%) compared to *Nlrp3*^*−/−*^ (38.0% ± 2.1%), *Pycard*^*−/−*^ (36.5% ± 0.6%), *Casp1*^*−/−*^ (37% ± 2.7%) and *Gsdmd*^*−/−*^ (37.2% ± 1.6%) mice. These data demonstrate the functional requirement of the NLRP3 inflammasome complex and this signaling cascade for AβOs-induced cytotoxicity.

### AβOs-induced degeneration requires RPE expression of P2RX7

**Fig. 3:**
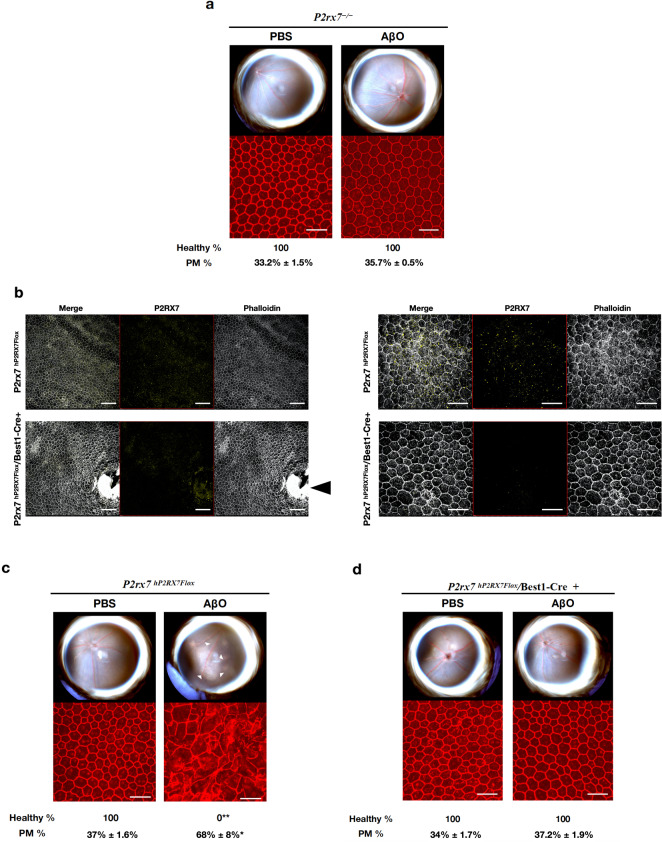
P2RX7 expression is required for AβOs-induced RPE degeneration. Eyes were treated with a single subretinal injection of 1 μM AβOs. Tissue was collected 7 days after injection. **a** *P2rx7*^*−/−*^ mice are protected from AβOs-induced RPE degeneration, *n* = 8. **b** Lower magnification (left panel) and higher magnification (right panel) of RPE flat mounts of *P2rx7*^*hP2RX7Flox*^ and *P2rx7*^*hP2RX7Flox*^*/*Best1-Cre+ mice stained with phalloidin (white) and P2RX7 (yellow) demonstrating reduction of P2RX7 signal in the RPE of *P2rx7*^*hP2RX7Flox*^*/*Best1-Cre+ mice compared to *P2rx7*^*hP2RX7Flox*^ mice. Black arrowhead points to the optic nerve of *P2rx7*^*hP2RX7Flox*^*/*Best1-Cre+ mice, where expression of P2RX persists in non-RPE tissue. **c** AβOs induced degeneration in *P2rx7*^*hP2RX7Flox*^ (*n* = 6) but not in *P2rx7*^*hP2RX7Flox*^*/*Best1-Cre+ mice (*n* = 8) (**d**). Representative images are shown. Fundus photographs, top row; Flat mounts stained for zonula occludens-1 (ZO-1; red), bottom row. Degeneration outlined by white arrowheads. Binary (Healthy %) and morphometric (PM, polymegethism (mean (SEM)) quantification of RPE degeneration is shown (Fisher’s exact test for binary; two-tailed *t*-test for morphometry; *P < 0.01, **P < 0.001). Loss of regular hexagonal cellular boundaries in ZO-1 stained flat mounts is indicative of degenerated RPE. Scale bars for lower magnification (100 μm) and higher magnification (50 μm)

Subretinal injection of AβOs did not induce degeneration in *P2rx7*^*−/−*^ mice (Fig. 3a and Supplementary Fig. 4), consistent with P2RX7 signaling lying upstream of the NLRP3 inflammasome.^35^ However, significant species heterogeneity exists between human and rodent P2RX7 in terms of immune activation and responses.^36^ In addition, *P2rx7*^*−/−*^ mice are reported to have partially functional P2X7R due to splice variants that evade inactivation.^37^ To overcome these two confounding issues, we tested *P2rx7*^*hP2RX7Flox*^/Best1-Cre+ mice, which we generated by interbreeding Best1-Cre+ mice with *P2rx7*^*hP2RX7Flox*^ mice, in which the mouse *P2rx7* gene locus was replaced with a floxed humanized *P2RX7* allele.^37^ We found that subretinal injection of AβOs induced RPE degeneration in *P2rx7*^*hP2RX7Flox*^/Best1-Cre+ mice, which expressed human P2X7R but did not induce RPE degeneration in *P2rx7*^*hP2RX7*^*/*Best1-Cre+ mice, in which P2RX7 expression is ablated in the RPE (Fig. 3b–d and Supplementary Fig. 4). Morphometric analysis of the RPE flat mounts revealed significantly higher (*P* < 0.001) polymegethism in *P2rx7*^*hP2RX7Flox*^ mice (68% ± 8.0%) compared to *P2rx7*^*−/−*^ (35.7% ± 0.5%) and *P2rx7*^*hP2RX7Flox*^/Best1-Cre+ mice (37.2% ± 1.9%). These findings provide evidence that AβOs induce human P2RX7 signaling and this is necessary for AβO-induced RPE degeneration.

### NRTIs and Kamuvudines inhibit AβOs-induced RPE degeneration

**Fig. 4:**
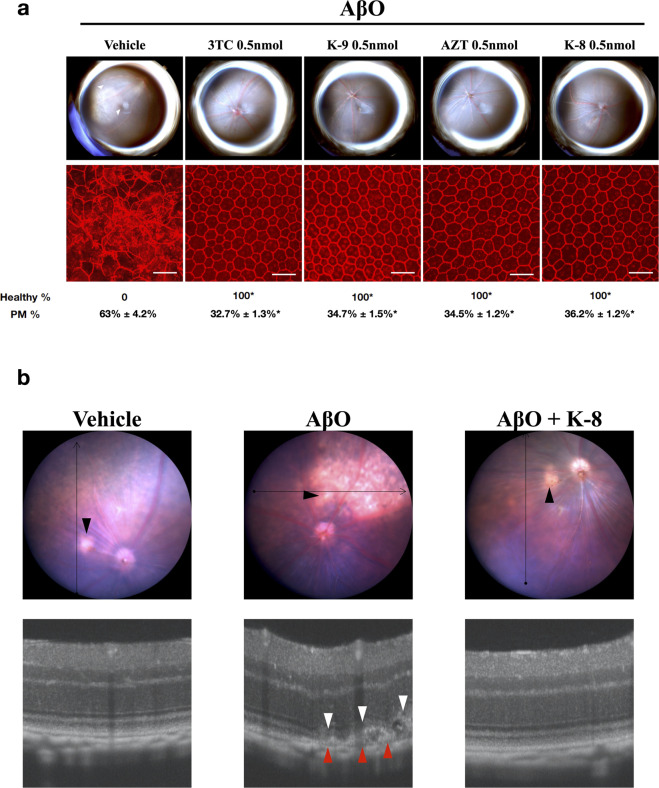
NRTIs and Kamuvudines inhibit AβOs-induced RPE degeneration. **a** Fundus photographs (top row) and flat mounts stained for zonula occludens-1 (ZO-1; red) (bottom row) of WT mice injected subretinally with 1 μM AβOs and intravitreously with vehicle or 0.5 nmol of NRTIs/Kamuvudines. Intravitreous administration of 3TC, K-9, AZT, or K-8 blocked AβOs induced RPE degeneration while the vehicle (PBS) did not. Degeneration outlined by white arrowheads. Representative images of *n* = 6–12. Binary (Healthy %) and morphometric (PM, polymegethism (mean (SEM)) quantification of RPE degeneration is shown (Fisher’s exact test for binary; two-tailed *t*-test for morphometry; *P < 0.001). Loss of regular hexagonal cellular boundaries in ZO-1 stained flat mounts is indicative of degenerated RPE. Scale bars (50 μm). **b** Image-guided spectral-domain optical coherence tomography 7 days after subretinal injection of vehicle (left), 1 μM AβOs (middle), and 1 μM AβOs plus intravitreous 0.5 nmol K-8 (right). Black arrowheads point to the injection site, red arrowheads point to RPE degeneration, and white arrowheads point to disruption of the photoreceptor outer segments

Next, we tested two classes of small molecule inflammasome inhibitors: NRTIs and Kamuvudines (modified NRTIs). We tested these drugs in a dose range where they block *Alu* RNA-induced RPE degeneration.^17,38^ We found that intravitreous administration of two NRTIs (lamivudine and zidovudine) or two Kamuvudines (2-ethyl-zidovudine and 3-methyl-lamivudine) blocked AβOs-induced RPE degeneration in a dose-dependent manner (Fig. 4a and Supplementary Figs. S5, S6). Morphometric analysis of the RPE flat mounts revealed significantly higher (*P* < 0.001) polymegethism in vehicle-treated mice (63.0% ± 4.2%) compared to mice treated with 3TC (32.7% ± 1.3%), K-9 (34.7% ± 1.5%), AZT (34.5% ± 1.2%), and K-8 (36.2% ± 1.2%). In addition, optical coherence tomography (OCT) imaging demonstrated disruption of the RPE and the photoreceptor outer segments after subretinal injection of AβOs that was prevented by intravitreous administration of K-8 (Fig. 4B). We next assessed retinal anatomy and function 4 weeks after intravitreous injection of K-8 at a dose ~25 times the therapeutic dose: we found, using histological examination and full-field electroretinography, that K-8 did not affect retinal morphology or function (Supplementary Fig. 7).

## Discussion

Our studies demonstrate that AβOs induce NLRP3 inflammasome priming, assembly, and activation and that AβOs-induced RPE degeneration is dependent on NLRP3. Further, we show that AβOs-induced RPE degeneration requires the expression of P2RX7 in the RPE. Finally, we identify two FDA-approved NRTIs, as well as their less-toxic Kamuvudine derivatives as inhibitors of AβOs-induced RPE degeneration. Coupled with our earlier demonstration that SINE RNA-induced RPE degeneration also requires NLRP3^11^ and P2RX7,^18^ our data argue that the P2RX7-NLRP3 signaling axis is a common checkpoint in multiple GA-relevant models of RPE degeneration. In addition, the ability of NRTIs and Kamuvudines to inhibit RPE degeneration by both SINE RNAs^17,38^ and AβOs enhances the rationale to test them in GA clinical trials.

GA is an example of a chronic, polygenic disease in which complex interplay between multiple genetic variants, environmental and lifestyle factors determine disease susceptibility, progression, and severity.^2,39^ As with several other age-related diseases, the development of successful therapeutic strategies for GA remains challenging and has been a futile endeavor thus far. The predominant reason for this challenge is the multi-factorial nature of the disease associated with multiple intrinsic and extrinsic stimuli or stressors.^39^ Despite varying initiating events, the invariant loss of RPE in GA supports the notion that this disease shares a common pathobiological course.^2^ Recently, there has been considerable emphasis on identifying commonly shared pathways driving disease progression in several age-related diseases.^40–42^ Chronic inflammation has been recognized as one of the tentative hallmarks that represent the common denominators of aging.^43^ Among the several mediators of inflammation, the NLRP3 inflammasome consistently takes a central position in aging as it is activated by a vast variety of aging-associated danger patterns.^44,45^

P2RX7-mediated NLRP3 inflammasome activation has been identified as a critical pathway necessary for RPE degeneration in several well-characterized models of GA.^18–20^ Here, we show that P2RX7-NLRP3 inflammasome activation is essential for RPE degeneration induced by AβOs, another well-recognized stressor implicated in the pathogenesis of GA. Dysregulated activation of the complement system is also implicated as a potential stressor in the pathogenesis of GA.^46–48^ Inflammasome genes are upregulated in human eyes with the complement factor H Y402H polymorphism,^49^ which is associated with increased risk of AMD.^50,51^ In addition, RPE cell culture models and animal models have also implicated inflammasome activation as an important driver of complement-induced RPE cytotoxicity.^52,53^ Interestingly C3a and C5a, critical components of the complement system that are elevated in human AMD eyes^54^ and putative drug targets for GA,^55^ activate the NLRP3 inflammasome,^53,54,56^ at least in part via P2RX7.^56^ Collectively, it is tempting to speculate that P2RX7-mediated NLRP3 inflammasome activation constitutes a unifying molecular cornerstone across diverse pathological pathways in GA.

NRTIs inhibit P2RX7 and block NLRP3 inflammasome activation independent of their ability to block reverse transcriptase.^17^ NRTIs are reported to have therapeutic potential against both forms of AMD, GA, and choroidal neovascularization, in animal models.^17,38,57^ In addition, clinical evidence has recently emerged that the use of NRTIs reduces the development of type 2 diabetes, which is inflammasome-driven.^58^ The efficacy of NRTIs and their non-toxic modifications, Kamuvudines, has been established in SINE RNA-induced RPE degeneration, a well-characterized model of GA.^17^ This study, by reporting the efficacy of NRTIs and Kamuvudines in blocking AβOs-induced NLRP3 activation and subsequent RPE degeneration provides a strong impetus to explore these modified NRTIs as possible drug candidates for treating GA, particularly as they have a high therapeutic index.

## Materials and methods

### Amyloid oligomer preparation

The methods employed to oligomeric Aβ preparations were modified from previously described reports.^59,60^ Human lyophilized, synthetic Aβ1–40 (BACHEM, Bubendorf, Switzerland) or Aβ 40-1 (BACHEM, Bubendorf, Switzerland) was suspended in 1,1,1,3,3,3-hexafluoro-2-propanol (HFIP) (Sigma-Aldrich, St. Louis, MO) and incubated at room temperature for 5 h to establish monomerization. The peptide was then aliquoted into microfuge tubes and evaporated overnight at room temperature. Complete removal of HPIF was ensured via a CentriVap Concentrator (Labconco, Kansas City, MO) for 1 h. The dried peptide was resuspended for 5 min at room temperature in 40–50 μl dimethylsulfoxide (DMSO) to ∼1 mM, bath-sonicated for 10 min, diluted to 100 μM with 1× Phosphate-buffered saline and vortexed for 10 s. Aggregation was allowed to proceed for 48 h at 4 °C with rocking. Following the 48 h incubation, oligomer preparations were centrifuged at 12,700 rpm for 15 min at 4 °C to remove fibrils. The presence of oligomers in the preparations was confirmed by western blotting (Supplementary Fig. 8). Samples were analyzed using NuPAGE Novex 4–20% Tris-Glycine gels (Invitrogen, Carlsbad, CA) and NativePAGE running buffer (Invitrogen, Carlsbad, CA). The semi-dry transfer was performed using a Trans-Blot Turbo Transfer System (BioRad, Hercules, CA). The membranes were incubated in primary antibody against amino acid residues 1–16 of Aβ (6E10, 1:1000, SIG-39320, Biolegend, SanDiego, CA) or against synthetic molecular mimic of soluble oligomers of Aβ (A11, 1:1000, AHB0052, ThermoFisher Scientific, Waltham, MA) overnight at 4 °C. After washing, incubation in secondary antibody (α-mouse IgG-HRP 1:5000, α-rabbit IgG-HRP 1:5000, Invitrogen) was performed for 1 h at room temperature. Subsequently, the membranes were visualized using a LI-COR Odyssey Clx Imaging system (LI-COR, Lincoln, NE) and analyzed using the Image Studio Lite Software (LI-COR, Lincoln, NE).

### Mice

All animal experiments were approved by the University of Virginia Institutional Animal Care and Use Committees and were performed in accordance with the Association for Research in Vision and Ophthalmology Statement for the Use of Animals in Ophthalmic and Visual Research. Both male and female mice between 6 and 10 weeks of age were used in the study. Wild-type C57BL/6J, Best1-Cre, R26-CAG-LSL-ASC-citrine, and *P2rx7*^*−/−*^ mice were obtained from The Jackson Laboratory. *Gsdmd*^*−/−*^ and *Pycard*^*−/−*^ mice^61,62^ described earlier were a generous gift from V.M Dixit (Genentech). *Nlrp3*^*−/−*^ and *Casp1*^*−/−*^ mice described earlier^63^ were a generous gift from G. Nunez (University of Michigan). *P2rx7*^*hP2RX7Flox*^ mice have been previously described^37^ (Supplementary Fig. 9 and Supplementary Table 1). *Nlrp3-*GFP mice described earlier^31,32^ were a generous gift from F. Martinon and P. Schneider. Conditional RPE-specific knockout mice were generated by interbreeding the “floxed mice” with Best1-Cre mice. For all procedures, anesthesia was achieved by intraperitoneal injection of 100 mg/kg ketamine hydrochloride (Ft. Dodge Animal Health) and 10 mg/kg xylazine (Phoenix Scientific), and pupils were dilated with topical 1% tropicamide and 2.5% phenylephrine (Alcon Laboratories).

### Synthesis of modified NRTIs/Kamuvudines

The Kamuvudines 3-methyl-3TC (3-Me-3TC) and 2-ethyl-AZT (2-Et-AZT), were synthesized as described previously.^17^

### Subretinal injections

Subretinal injections (SRI) (1 μl) of AβOs (1 μM) were performed in mice using a 35-gauge needle (Ito Co. Fuji, Japan) as described earlier.^64^

### Drug treatments

Intravitreous administration (0.5 μl) dose ranging from 0.1 to 0.5 nmol of AZT, 3TC (SelleckChem, Houston, TX, USA), K-8, K-9, or vehicle (PBS) was performed using a 35-gauge needle (Ito Co. Fuji, Japan) immediately after the subretinal injection. For the toxicity studies, 12 nmol of K-8 was injected intravitreously in the right eye and PBS was injected in the left eye.

### Fundus photography

Fundus imaging of dilated mouse eyes was performed using a TRC-50 IX camera (Topcon) linked to a digital imaging system (Sony).

### Assessment of RPE degeneration

Seven days after SRI, RPE health was assessed by fundus photography and immunofluorescence staining of zonula occludens-1 (ZO-1) on RPE flat mounts (whole mount of posterior eye cup containing RPE and choroidal layers). Mouse RPE and choroidal flat mounts were fixed with 2% paraformaldehyde, stained with rabbit polyclonal antibodies against mouse ZO-1 (1:100, Invitrogen), and visualized with Alexa-594 (Invitrogen). All images were obtained by confocal microscopy (model A1R Nikon confocal microscope system, Nikon). The injection site was identified by the characteristic stellate pattern of the RPE at the injection site (Supplementary Fig. 10). The injection site and the surrounding area are first identified using lower magnification, followed by the acquisition of images using higher magnification. Imaging was performed by an operator blinded to the group assignments. RPE degeneration was quantified based on zonula occludens (ZO)-1-stained flat-mount images using two strategies: (1) Binary Assignment: RPE health was assessed based on the presence or absence of morphological disruption in RPE flat mounts by two independent raters who were masked to the group assignments.^13^ Both raters deemed 100% of images as gradeable. (inter-rater agreement = 100%; Pearson *r*^2^ = 1, *P* < 0.0001). (2) Semi-automated cellular morphometry analysis for hexagonally packed cells was performed by three masked graders as previously described. For this analysis, microscopy images of the RPE were captured and transmitted in deidentified fashion to the Doheny Image Reading and Research Lab (DIRRL). All images were rescaled to 304 × 446 pixels to permit importation into the Konan CellCheck software (Ver. 4.0.1), a commercial U.S. FDA-cleared software that has been used for clinical trials. RPE cell metrics were generated by three certified reading center graders in an independent, masked fashion. Once the cell centers were defined, the software automatically generated the polymegethism values.

### Immunostaining for NLRP3-GFP and ASC specks

For NLRP3-GFP visualization, AβOs were injected into the subretinal space in *Nlrp3*-GFP reporter mice followed by immediate intravitreous administration (0.5 μl) of the Caspase-1 inhibitor (Ac-YVAD-fmk, InvivoGen) to prevent distortion of RPE cellular architecture without influencing inflammasome assembly. Forty-eight hours after SRI, RPE flat mounts were prepared and fixed as described above. Fixed RPE flat mounts were stained with Alexa-594 conjugated rabbit polyclonal antibodies against mouse ZO-1 (1:100, Invitrogen) and conjugated anti-GFP polyclonal Alexa-488 antibodies (1:100, Invitrogen). For ASC speck visualization, AβOs were injected into the subretinal space in ASC-Citrine^Flox^/Best1-Cre+ mice followed by immediate intravitreous administration (0.5 μl) of the Caspase-1 inhibitor (Ac-YVAD-fmk, InvivoGen) to prevent distortion of RPE cellular architecture without influencing inflammasome assembly. At 48 h after SRI, RPE flat mounts were prepared and fixed as described above. The fixed RPE flat mounts were stained with Alexa-594 conjugated rabbit polyclonal antibodies against mouse ZO-1 (1:100, Invitrogen) and rabbit polyclonal anti-Cre recombinase antibody (1:100, Abcam) followed by a goat anti-rabbit Alexa-647 antibody (1:200, Invitrogen). All images were obtained by confocal microscopy (model A1R Nikon confocal microscope system, Nikon). Imaging was performed by an operator blinded to the group assignments.

### Immunostaining for humanized P2RX7

Eyes from *P2rx7*^*hP2RX7Flox*^ mice and *P2rx7*^*hP2RX7Flox*^ mice crossed with Best1-Cre mice were collected and fixed as described above. The RPE flat mounts were stained with Dylight phalloidin 650 (1:10, Cell Signaling) and a rabbit polyclonal anti-P2RX7 (extracellular) antibody (1:100, Alomone Labs), followed by a goat anti-rabbit Alexa-555 antibody (1:200, Invitrogen).

### Histology

For hematoxylin and eosin staining, fresh, unfixed mouse eyes were embedded in Optimal Cutting Temperature Compound (Fisher), frozen in isopentane precooled by liquid nitrogen, and cryosectioned at 10 μm.

### Electroretinography (ERG)

ERG was performed 4 weeks after the subretinal injection. Mice (*n* = 4–6) were dark-adapted overnight before the experiments and anesthetized as described above. The pupils were dilated with tropicamide (1%) and phenylephrine (2.5%) eye drops. ERG was recorded using a Ganzfeld ERG (Phoenix laboratories). Scotopic combined responses were obtained using the LabScribe software (Phoenix Laboratories) under dark-adapted conditions (no background illumination, 0 cd/m^2^) in response to white-flash stimuli ranging from −1.7 to 1.0 log cd s/m^2^ with twenty responses averaged for each stimulus.

### Statistics

The binary readouts of RPE degeneration (i.e., presence or absence of RPE degeneration on fundus and ZO-1-stained flat-mount images) were analyzed using Fisher’s exact test. Cell morphometry data were assessed using a Student *t*-test. *P* values < 0.05 were deemed statistically significant.

## Supplementary information

Supplementary Material

## Data Availability

The authors confirm that the data supporting the findings of this study are available within the article and its supplementary materials.
