# Supplementary material for: Nucleoside reverse transcriptase inhibitors and Kamuvudines inhibit amyloid-β induced retinal pigmented epithelium degeneration
Source: Signal Transduct Target Ther. 2021 Apr 14;6:149. doi: 10.1038/s41392-021-00537-z (PMC8044134; doi:10.1038/s41392-021-00537-z)
Supplement: Supplementary file 1 — Supplementary Material [file 41392_2021_537_MOESM1_ESM.pdf]

Supplementary Materials for

Nucleoside reverse transcriptase inhibitors and Kamuvidines inhibit amyloid-β induced retinal pigmented epithelium degeneration

Siddharth Narendran[1,2,3], Felipe Pereira[1,2,4], Praveen Yerramothu[1,2,], Ivana Apicella[1,2], Shao-bin Wang[1,2], Kameshwari Ambati[1,2], Shuichiro Hirahara[1,2], Younghee Kim[1,2], Meenakshi Ambati[1,2,5], Vidya L. Ambati[5], Peirong Huang[1,2], Akhil Varshney[1,2,], Yosuke Nagasaka[1,2,], Shinichi Fukuda[1,2,], Kirstie L. Baker[6], Kenneth M. Marion[6], Jan M. Deussing[7], Srinivas R. Sadda[6,8], Bradley D. Gelfand[1,2,9] and Jayakrishna Ambati[1,2,10,11]

Correspondence: Jayakrishna Ambati (ja9qr@virginia.edu)

This PDF file includes:

Figures S1 to S10
Table S1

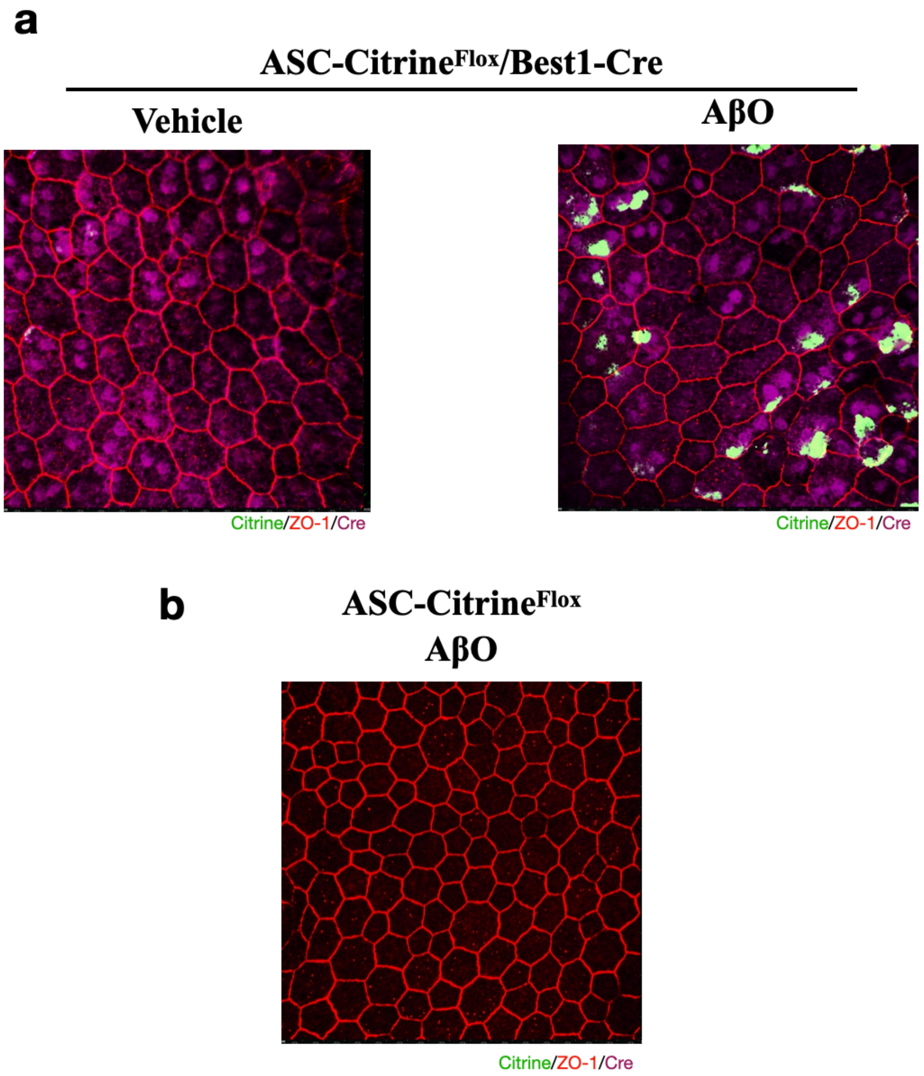

Fig. S1 Subretinal injection of AβOs induces ASC speck formation in vivo. Mice were treated with subretinal injection of 1 μM AβOs, followed by intravitreous injection of caspase-1 inhibitor to prevent RPE degeneration and thereby preserve the ability to visualize inflammasome assembly. Tissues were collected at 48 h after injection. (**a**) RPE flat mount, stained for zonula occludens-1 (ZO-1; red), of ASC-Citrine^{Flox}/Best1 Cre+ mice injected with vehicle or AβOs illustrates the mosaic expression of Cre (purple) in these mice and the presence of the citrine signal (green) only in cells expressing Cre. (**b**) RPE flat mount of ASC-Citrine^{Flox} mice injected with AβOs illustrates the absence of Cre staining (purple) and of the citrine signal (green) in these mice.

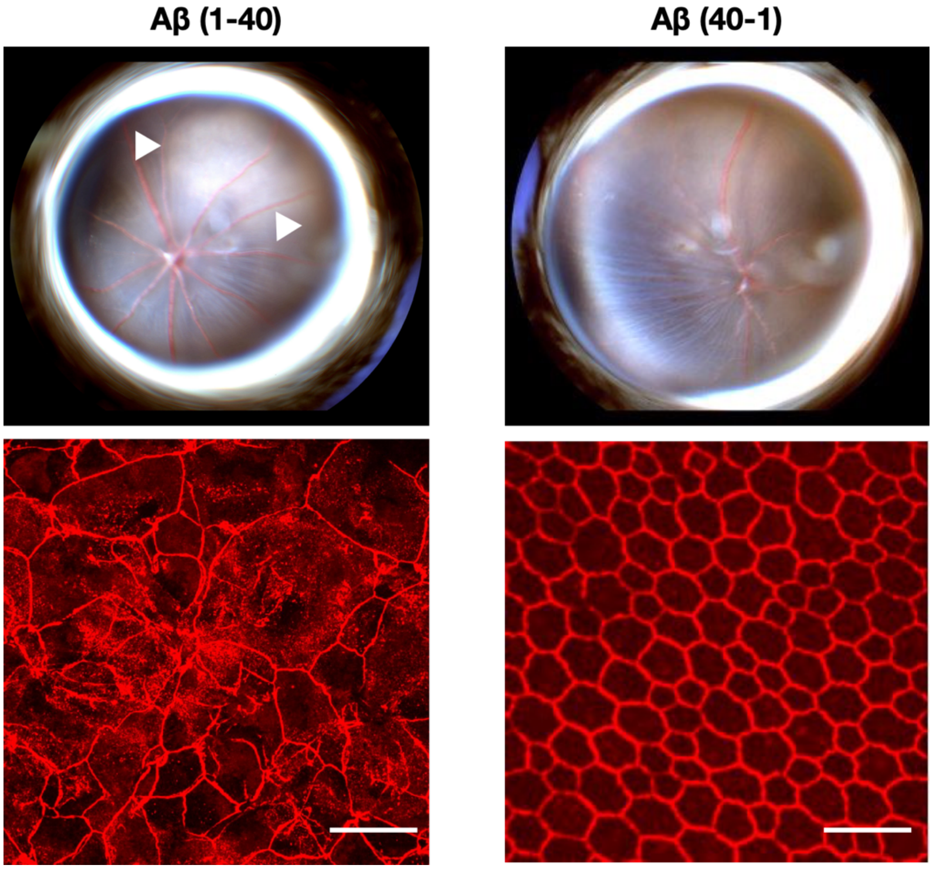

Fig. S2 Soluble oligomers of Aβ 1-40 and not Aβ 40-1 induce RPE degeneration. Mice were treated with subretinal injection of 1 μM of the Aβ 1-40 peptide (left) or the reverse peptide Aβ 40-1 (right). Oligomers of Aβ 1-40 induced RPE degeneration whereas Aβ 40-1 oligomers did not. Fundus photographs, top row; Flat mounts stained for zonula occludens-1 (ZO-1; red), bottom row. Degeneration outlined by white arrowheads. Representative images of n = 4-6. Loss of regular hexagonal cellular boundaries in ZO-1 stained flat mounts is indicative of degenerated RPE. Scale bars (50 μm).

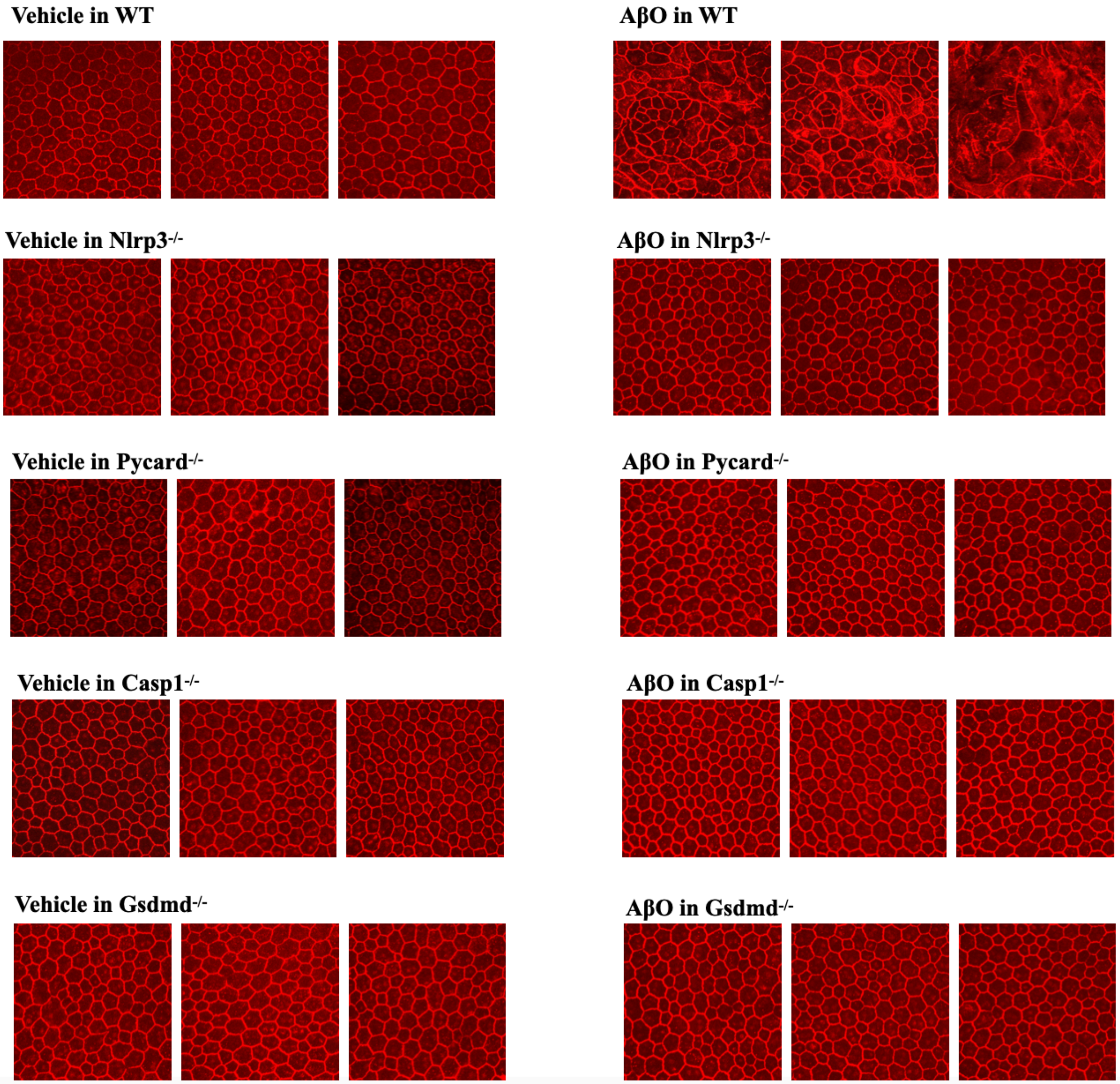

Fig. S3 AβOs-induced RPE degeneration requires *Nlrp3*, *Casp1*, *Pycard*, and *Gsdmd*. RPE flat mounts stained for zonula occludens-1 (ZO-1; red) show that subretinal injection of AβOs, but not PBS, induces RPE degeneration in WT mice but not in mice lacking *Nlrp3*, *Casp1*, *Pycard*, or *Gsdmd*. Loss of regular hexagonal cellular boundaries in ZO-1 stained flat mounts is indicative of degenerated RPE.

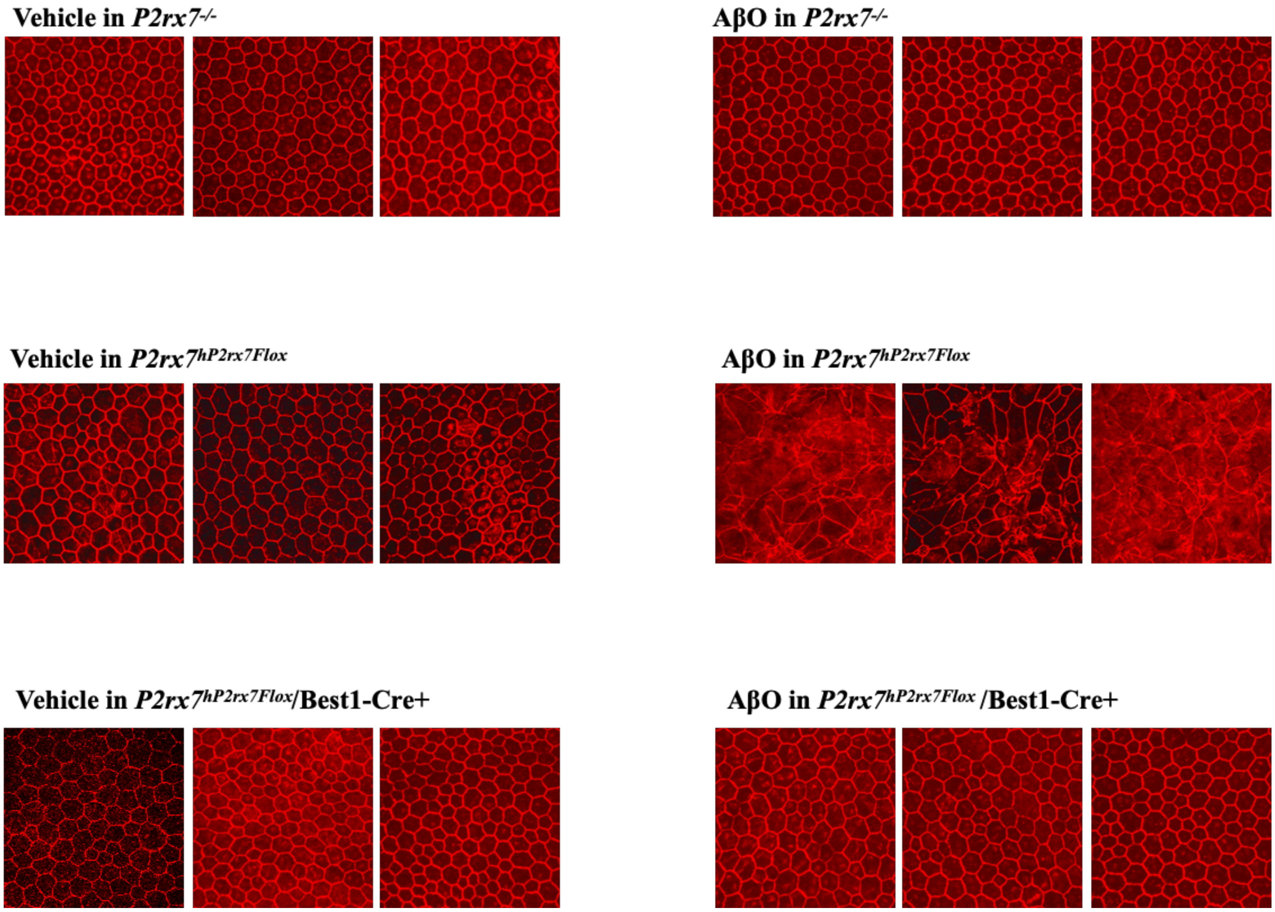

Fig. S4 AβOs-induced RPE degeneration requires *P2rx7* and human P2RX7 supports AβOs-induced RPE degeneration. RPE flat mounts stained for zonula occludens-1 (ZO-1; red) show that subretinal injection of AβOs did not induce RPE degeneration in $P2rx7^{-/-}$ mice (top row). AβOs induced RPE degeneration in $P2rx7^{hP2RX7Flox}$ mice, which express human *P2RX7* in place of the deleted mouse *P2rx7* gene locus (middle row). Loss of regular hexagonal cellular boundaries in ZO-1 stained flat mounts is indicative of degenerated RPE. AβOs did not induce RPE degeneration in $P2rx7^{hP2RX7Flox}$/Best1-Cre+ mice, in which human *P2RX7* has been conditionally ablated in the RPE (bottom row).

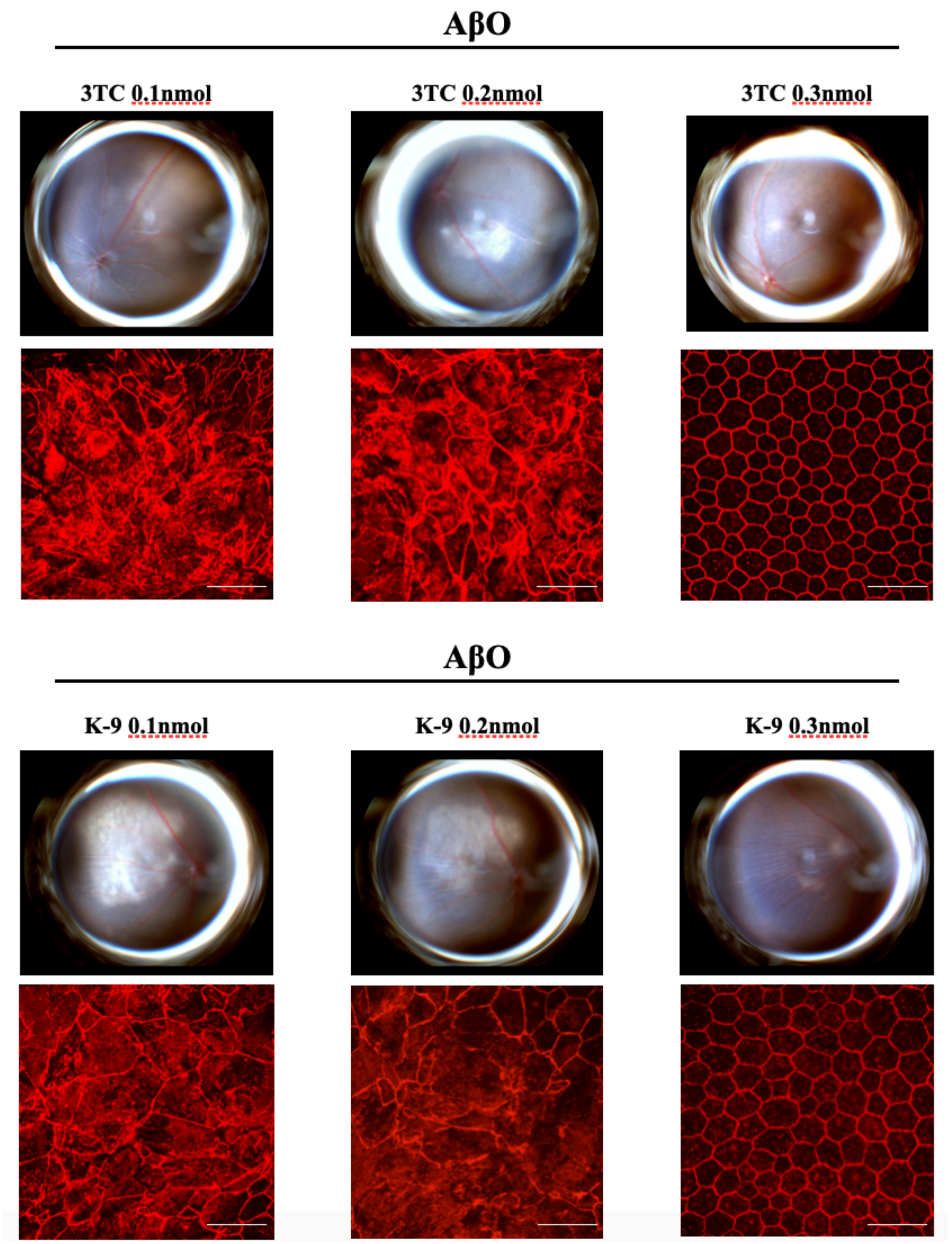

Fig. S5 Dose curve assessment of the NRTI lamivudine (3TC) and the modified NRTI K-9. Subretinal injection of 1 µM AβOs was followed by intravitreous injection of 0.1 nmol, 0.2 nmol or 0.3 nmol of 3TC or K-9. Fundus photographs (top row) and RPE flat mounts stained for zonula occludens-1 (ZO-1; red) (bottom row) of WT mice (n=4 per group). Loss of regular hexagonal cellular boundaries in ZO-1 stained flat mounts is indicative of degenerated RPE. Scale bars (50 µm).

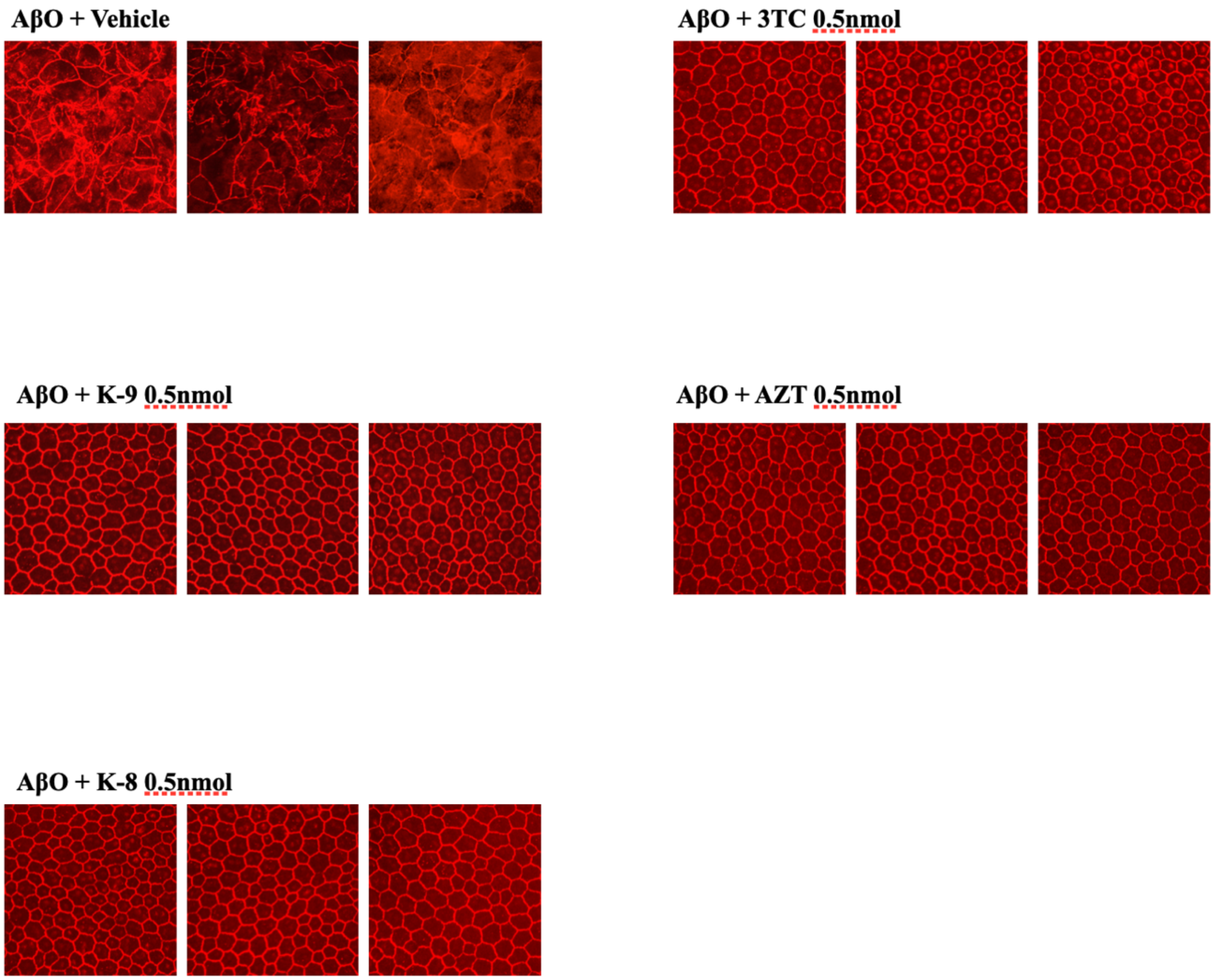

Fig. S6 NRTIs and Kamuvudines block AβO-induced RPE degeneration. RPE flat mounts stained for zonula occludens-1 (ZO-1; red) show that AβO-induced RPE degeneration in WT mice was blocked by intravitreous injection of the NRTIs 3TC or AZT or injection of the Kamuvudines K-8 or K-9.

a

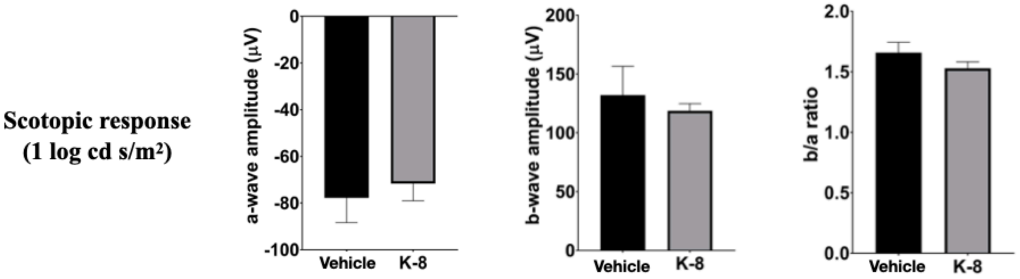

b

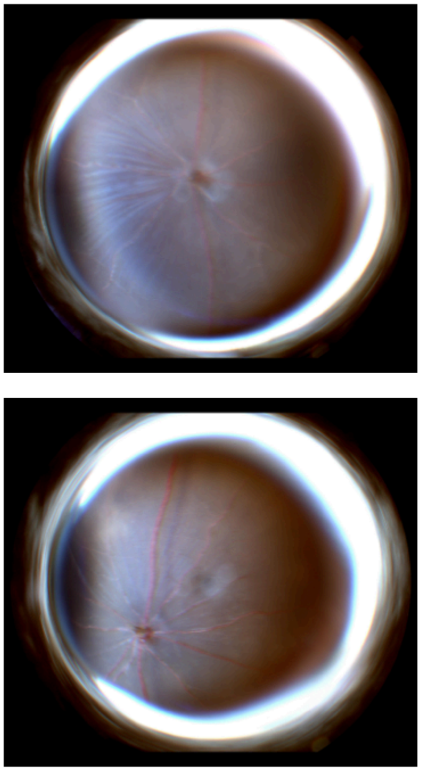

c

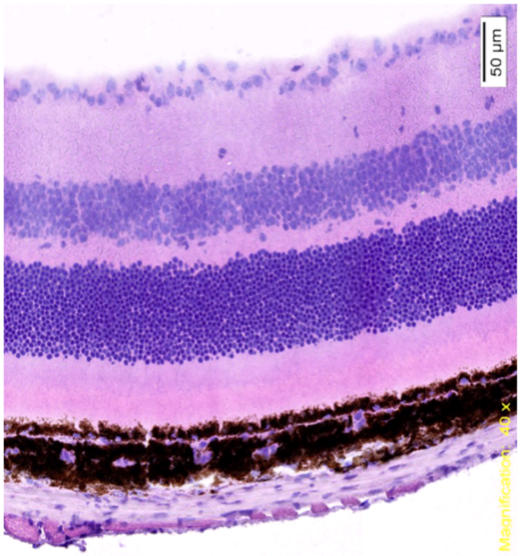

Fig. S7 K-8 does not induce intraocular toxicity. Scotopic full-field electroretinography (ERG), color fundus photography, and histology evaluations at 4 weeks after intravitreous injection of high-dose K-8 (12 nmol) or vehicle in WT mice. (**a**) ERG a-wave and b-wave amplitudes and b/a ratios were not significantly different between vehicle-treated and K-8-treated eyes. (**b**) Fundus images show normal retinal appearance and clear vitreous humor. (**c**) Hematoxylin- and eosin-stained retinal sections show normal neuroretinal, RPE, and choroidal morphology. N≥5. Error bars represent SEM.

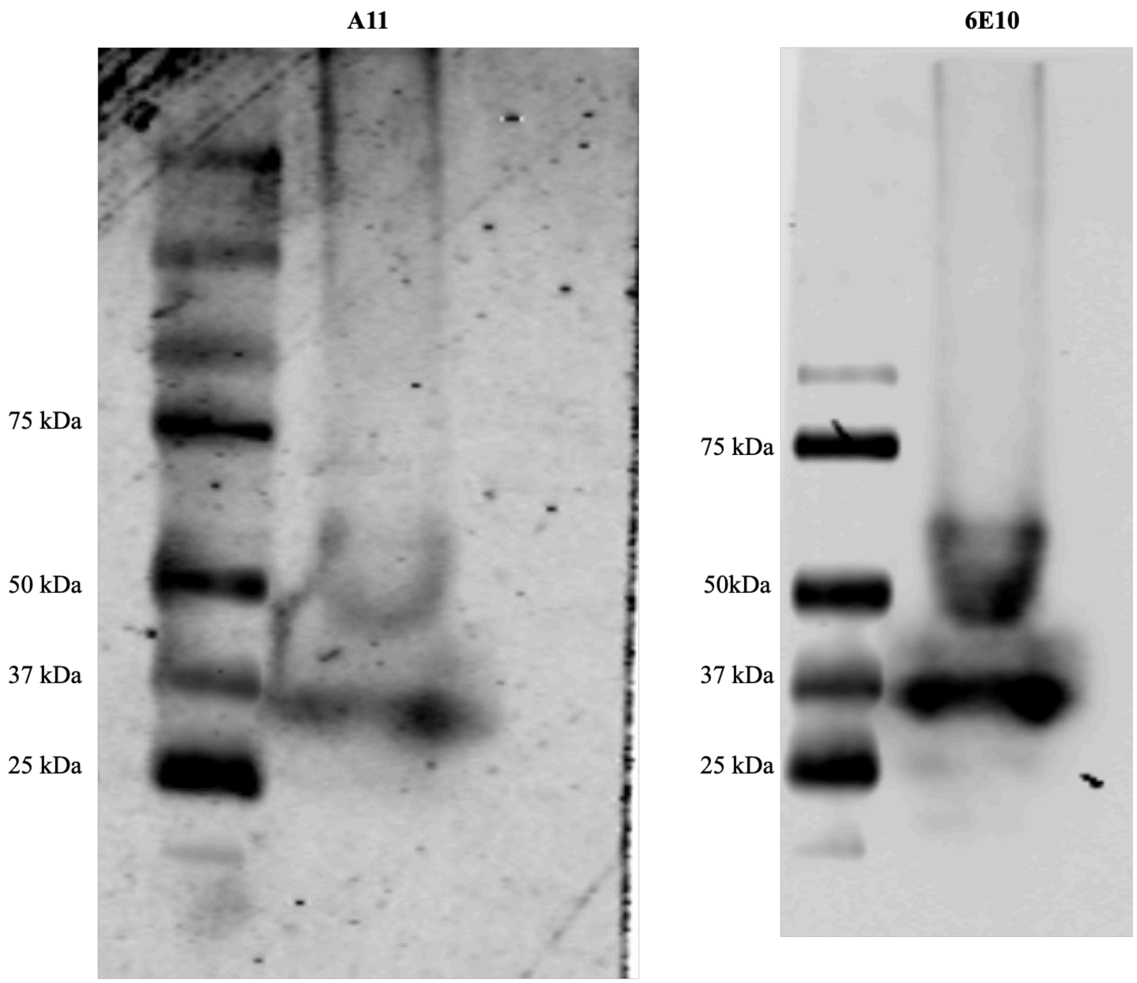

Fig. S8 Confirmation of Amyloid β oligomer formation. Western blot analysis of AβOs. AβOs were resolved on polyacrylamide gels under native running conditions and probed with A11 and 6E10 antibodies as indicated in the top panel.

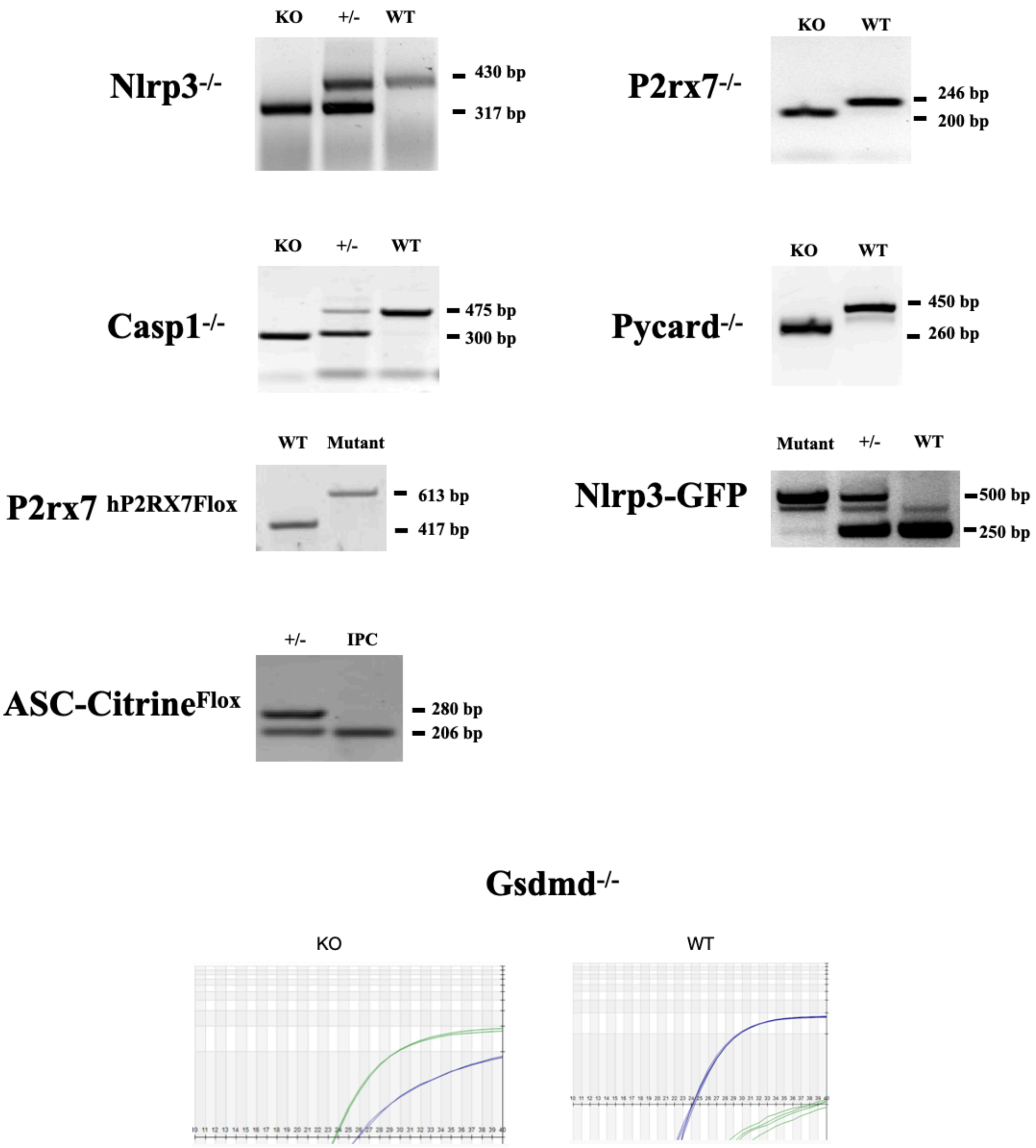

Fig. S9 Confirmation of gene ablation in transgenic mice. Genotyping performed by PCR of *Nlrp3*$^{-/-}$, *Pycard*$^{-/-}$, *Casp1*$^{-/-}$, *P2rx7*$^{-/-}$, *Gsdmd*$^{-/-}$, *P2rx7*^{hP2rx7Flox}, *Nlrp3*-GFP, and ASC-Citrine^{Flox} mice. Wild-type (WT), heterozygous (+/−), knockout (KO), and mutant allele with the respective size are shown. *Gsdmd*$^{-/-}$ mice were genotyped by Transnetyx Inc. IPC, internal positive control.

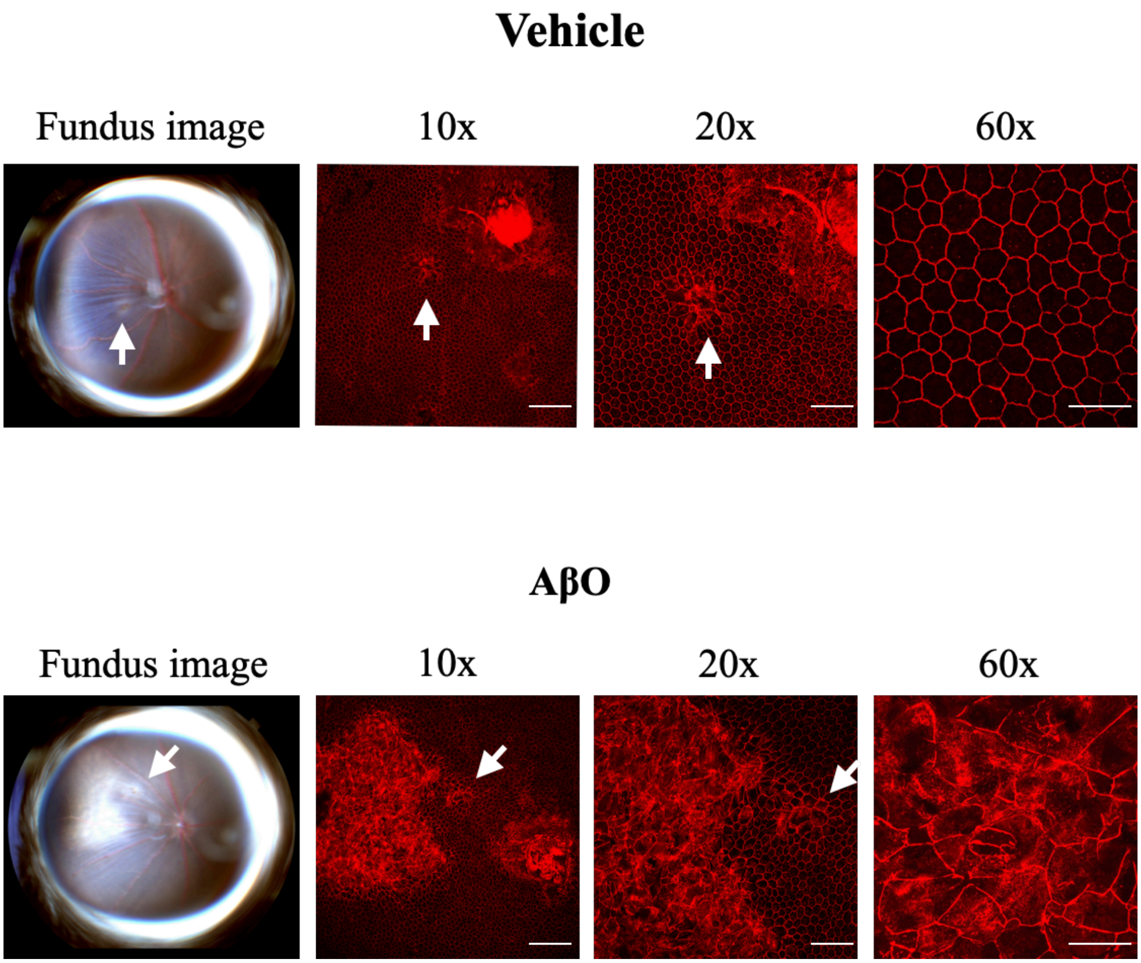

Fig. S10 Identification of the injection site in mouse RPE flat mounts. Subretinal injection of vehicle (top) or AβOs (lower) showing the fundus image (left), ZO-1 immunostaining (red) with lower (10× and 20×) and higher magnification (60×) of the same eye. The injection site is identified by a stellate pattern in the RPE (white arrow), which corresponds to the region where the needle touches the RPE. The surrounding area is examined, and higher magnification images are acquired and analyzed. Scale bars, 10× (200 μm), 20× (100 μm), 60× (50 μm).

Table S1. PCR primers used for genotyping

Name	5'-3' sequence
Nlrp3-F	CGGTGGTTGCTAGGAGATGG
Nlrp3-R	ATCGCCTTCTTGACGAGTTC
Pycard-1	CTAAGCACAGTCATTGTGAGCTCC
Pycard-2	CTAGTTTGCTGGGGAAAGAAC
Pycard-3	AAGACAATAGCAGGCATGCTGG
Casp1-F	GAGACATATAAGGGAGAAGGG
Casp1-R	TGCTAAAGCGCATGCTCCAGACTG
Gsdmd-F	GGGAACATTCAGGGCAGAGT
Gsdmd-R	CAGCCCCACCAGAAATTTTCC
Mouse P2rx7-F	TATACTGCCCCTCGGTCTTG
Mouse P2rx7-R	GCCAGAGGCCACTTGTGTAG
Human P2rx7-1	AGACTCTCACCAGCAGCAGCTC
Human P2rx7-2	GCCAAGCATTCTACCAGTTGAGC
Human P2rx7-3	CAGGATGTTTCTCGTGGTGTAG
Cre-F	ATGCCCAAGAAGAAGAGGAAGGTGTCCA
Cre-R	TGGCCCAAATGTTGCTGGATAGTTTTTA
Nlrp3-gfp-1	AAGTCGTGCTGCTTCATGT
Nlrp3-gfp-2	TCAAGCTAAGAGAACTTTCTG
Nlrp3-gfp-3	ACACTCGTCATCTTCAGCA
Asc f/f-F	CTTGGGTGGAGAGGCTATTC
Asc f/f-R	AGGTGAGATGACAGGAGATC
